# Incidence and Predictors of High-Grade AV Block in Patients With Initially Unchanged Electrocardiogram After TAVR

**DOI:** 10.1016/j.jscai.2025.103666

**Published:** 2025-05-27

**Authors:** Mohamed Elhadi, Mohamad S. Alabdaljabar, Conor Lane, Abhishek J. Deshmukh, Rajiv Gulati, Yong-Mei Cha, Mackram F. Eleid

**Affiliations:** Division of Interventional Cardiology, Department of Cardiovascular Medicine, Mayo Clinic, Rochester, Minnesota

**Keywords:** aortic stenosis, complete heart block, conduction disease, high-grade atrioventricular block, permanent pacemaker implantation, transcatheter aortic valve replacement

## Abstract

**Background:**

High-grade atrioventricular block (HAVB) is common after transcatheter aortic valve replacement (TAVR). We compared patients with baseline conduction disease that is unchanged on the immediate post-TAVR echocardiogram (ECG) to patients with normal baseline and post-TAVR ECG (control group).

**Methods:**

Consecutive patients who underwent TAVR at Mayo Clinic (Rochester, Minnesota) between February 2012 and December 2021 were retrospectively reviewed.

**Results:**

In total, 1069 patients were included in the study: 825 controls, 44 with isolated PR of >240 milliseconds, 93 with left bundle branch block (LBBB), and 107 with right bundle branch block (RBBB). Early HAVB (<24 hours post-TAVR) occurred more frequently in the RBBB group compared with controls (11.2% vs 0.6%; *P* < .001). Early HAVB incidence was similar between the control, isolated PR >240, and LBBB groups (0.6%, 0%, and 1.1%, respectively). Delayed HAVB (>24 hours post-TAVR) was most frequent in the RBBB group (6.5% vs 1.5%; *P* < .001), with higher incidence also observed in PR >240 and LBBB groups compared with that in control (4.5% vs 1.5%; *P* = .14; and 4.3% vs 1.5%; *P* = .06, respectivley). Most of HAVB events in the control, isolated PR >240 and LBBB groups were delayed.

**Conclusions:**

Despite no immediate change in post-TAVR ECG, 17.8% of patients with preexisting RBBB developed HAVB, mostly within 24 hours. This emphasizes the need for inpatient monitoring for at least 24 hours in this group. Conversely, in patients with isolated PR >240 milliseconds and LBBB, the incidence of HAVB was relatively low (5%), with the majority occurring after 24 hours. Potentially, same-day discharge with ambulatory ECG monitoring may be suitable for these patients.

## Introduction

Transcatheter aortic valve replacement (TAVR) is an established treatment for patients with severe aortic stenosis. The expansion of TAVR to low-risk patients alongside the advancements in experience and devices used has led to a reduction in the majority of periprocedural complications and deaths.^1^ Despite reductions in rates of high-grade atrioventricular block (HAVB) with high implantation techniques,^2^ conduction disease requiring permanent pacemaker (PPM) implantation continues to be the most common complication.^3^^,^^4^ In patients without HAVB immediately after valve deployment, the electrocardiogram (ECG) and telemetry monitoring are used for risk stratification and to identify whether patients are suitable for early discharge, extended monitoring, or prophylactic PPM. Although many procedural and patient factors impact the incidence of HAVB post-TAVR, right bundle branch block (RBBB) is the strongest predisposing risk factor.^5^^,^^6^ The presence of preexisting left bundle branch block (LBBB) or first-degree atrioventricular (AV) block have been identified as variable predictors of HAVB.^7^^,^^8^ Thus far, to our knowledge, there are no studies specifically investigating outcomes in patients with no change in the ECG post-TAVR. These factors lead to heterogeneity in the management of conduction disease post-TAVR, which may contribute to disparities in PPM implantation rates between centers.^9^ Accordingly, we aimed to conduct a study to investigate patients with baseline conduction disease that remained unchanged on the ECG immediately after valve deployment and compare them with patients with completely normal baseline and post-TAVR ECG.

## Methods

Consecutive patients who underwent TAVR at Mayo Clinic (Rochester, Minnesota) between February 2012 and December 2021 were retrospectively reviewed. Patients with previous PPM or implantable cardioverter-defibrillator, intraprocedural death, or valve-in-valve TAVR were excluded. Before TAVR, all patients were evaluated by the heart team with a baseline ECG and echocardiogram. TAVR procedures were performed as previously described.^10^ Most patients had a temporary balloon-tipped right ventricular pacemaker placed for rapid ventricular pacing that was removed before the patients left the catheterization laboratory. Continuous telemetry monitoring was performed intraprocedurally and continued for at least 24 hours in all patients. A 12-lead ECG was performed immediately post-TAVR in all patients. In this study, we included all patients with unchanged conduction by ECG before and after TAVR. We defined unchanged conduction as patients who had no new abnormalities on their post-TAVR ECG. Patients who had worsening of a baseline conduction abnormality were also labeled as unchanged for the purposes of our analysis. For example, a patient with baseline LBBB who had worsening of their QRS duration but no other new abnormality such as first-degree heart block would have been included in the analysis.

**Figure 1:**
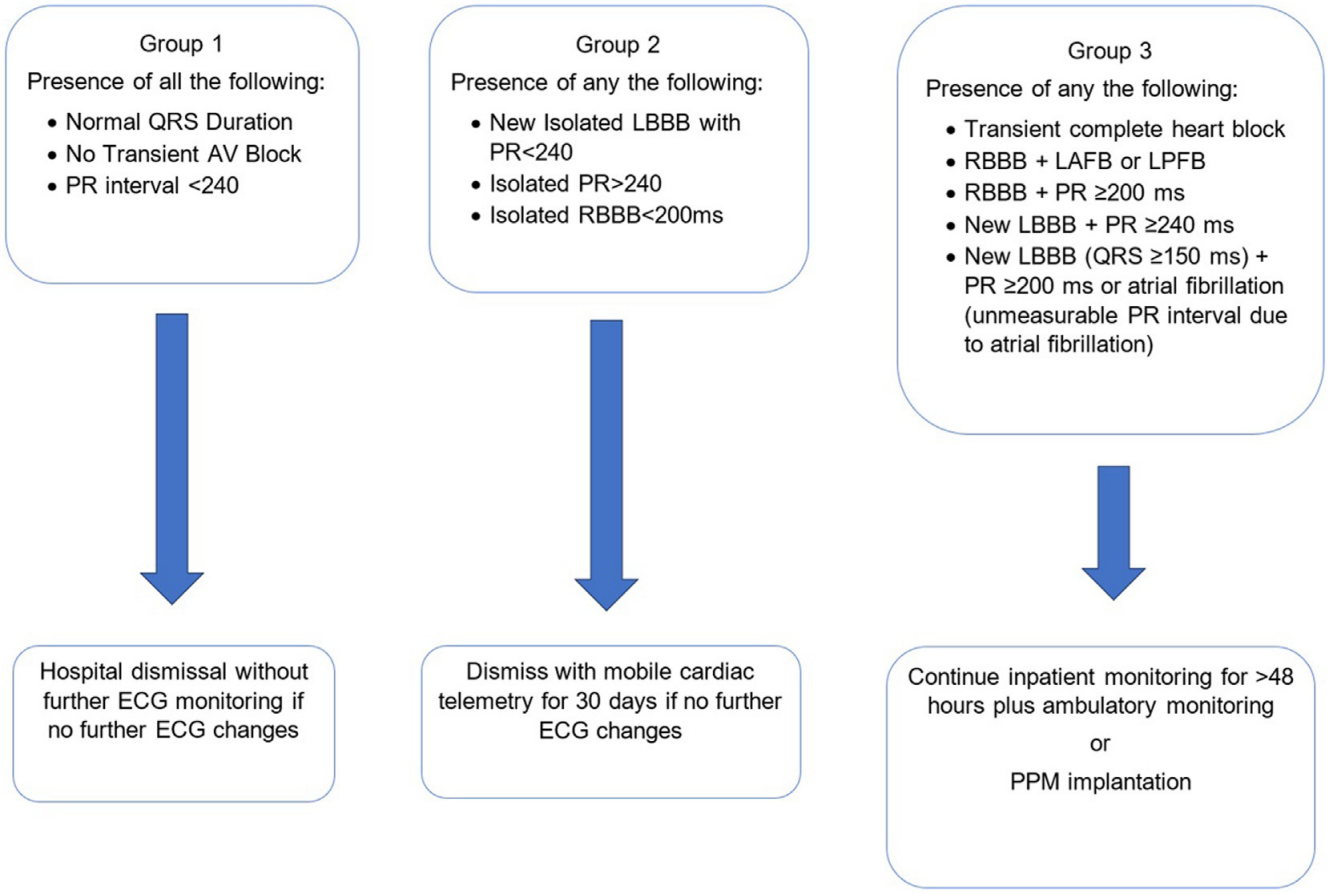
**Mayo Clinic post-TAVR conduction disease protocol.** The protocol uses the post-TAVR ECG to guide the decision to discharge the patient without further ECG monitoring, with a 30-day ambulatory ECG monitor or PPM implantation before discharge. AV, atrioventricular; ECG, electrocardiogram; LAFB, left anterior fascicular block; LBBB, left bundle branch block; LPBP, left posterior fascicular block; PPM, permanent pacemaker; RBBB, right bundle branch block; TAVR, transcatheter aortic valve replacement.

Our current institutional practice protocol of managing conduction disease post-TAVR divides patients into 3 categories based on ECG findings post-TAVR (Figure 1). This protocol was developed through a multidisciplinary approach, evolving throughout the institutional experience. The protocol guides the decision to discharge patients without further ECG monitoring, with a 30-day ambulatory ECG monitor or PPM implantation before discharge.

The patients in our study were divided into 4 groups. The control group included patients who had unchanged normal ECG after TAVR, including patients with a PR interval of <240 milliseconds. The other 3 groups were patients with the following unchanged baseline conduction abnormalities after TAVR: isolated PR interval >240 milliseconds, LBBB, and RBBB. In the RBBB group, patients with concomitant baseline left anterior fascicular block (LAFB) or left posterior fascicular block (LPFB) were included. We have excluded patients with sole preexisting and unchanged LAFB and LPFB due to low number of patients in both groups.

The Mayo Clinic Institutional Review Board approved the study, and no informed consent was required. The authors have full access to all data in the study and take responsibility for its integrity and the data analysis.

### Study end points and definitions

The primary outcome was the development of HAVB within 30 days. This was further stratified into early (between the time of the first postprocedural ECG and 24 hours) or delayed (between 24 hours and 30 days). The diagnosis of HAVB was determined by the presence of a minimum of 2 consecutive nonconducted P waves for patients in sinus rhythm or bradycardia <50 beats per minute with fixed rate for patients with atrial fibrillation or flutter. Given that all patients in the study had the same baseline ECG findings immediately post-TAVR, HAVB was detected on subsequent ECGs, telemetry monitoring, or 30-day ambulatory ECG monitoring. Baseline clinical and procedural characteristics were reported. The secondary outcomes included PPM implantation, transient intraprocedural heart block, 30-day mortality, and 1-year mortality. Transient intraprocedural heart block was defined as HAVB, which developed shortly after valve deployment with recovery of native conduction before transfer to a monitored bed. In patients who received PPM, the percentage right ventricular pacing at 1 to 2 months interrogation was reported. A subgroup analysis of patients with RBBB was completed to identify predictors of HAVB in this group.

### Statistical analysis

Descriptive statistics were used to analyze baseline characteristics, procedural characteristics, and outcomes. Categorical variables were expressed as n (%) and continuous variables as median and interquartile range. Continuous and categorical variables were compared between the control group and each of the other 3 groups, with statistical significance being evaluated using the Mann-Whitney *U* test and χ^2^ test, respectively. A statistical significance level of *P* < .05 was used. Logistic regression was used in the RBBB group to assess risk factors associated with HAVB. The number of variables in the multivariable model was limited to be less than the number of events divided by 10 (to avoid model overfitting). Statistical analyses were performed using IBM SPSS Statistics for Windows, version 29 (IBM Corp).

## Results

**Figure 2:**
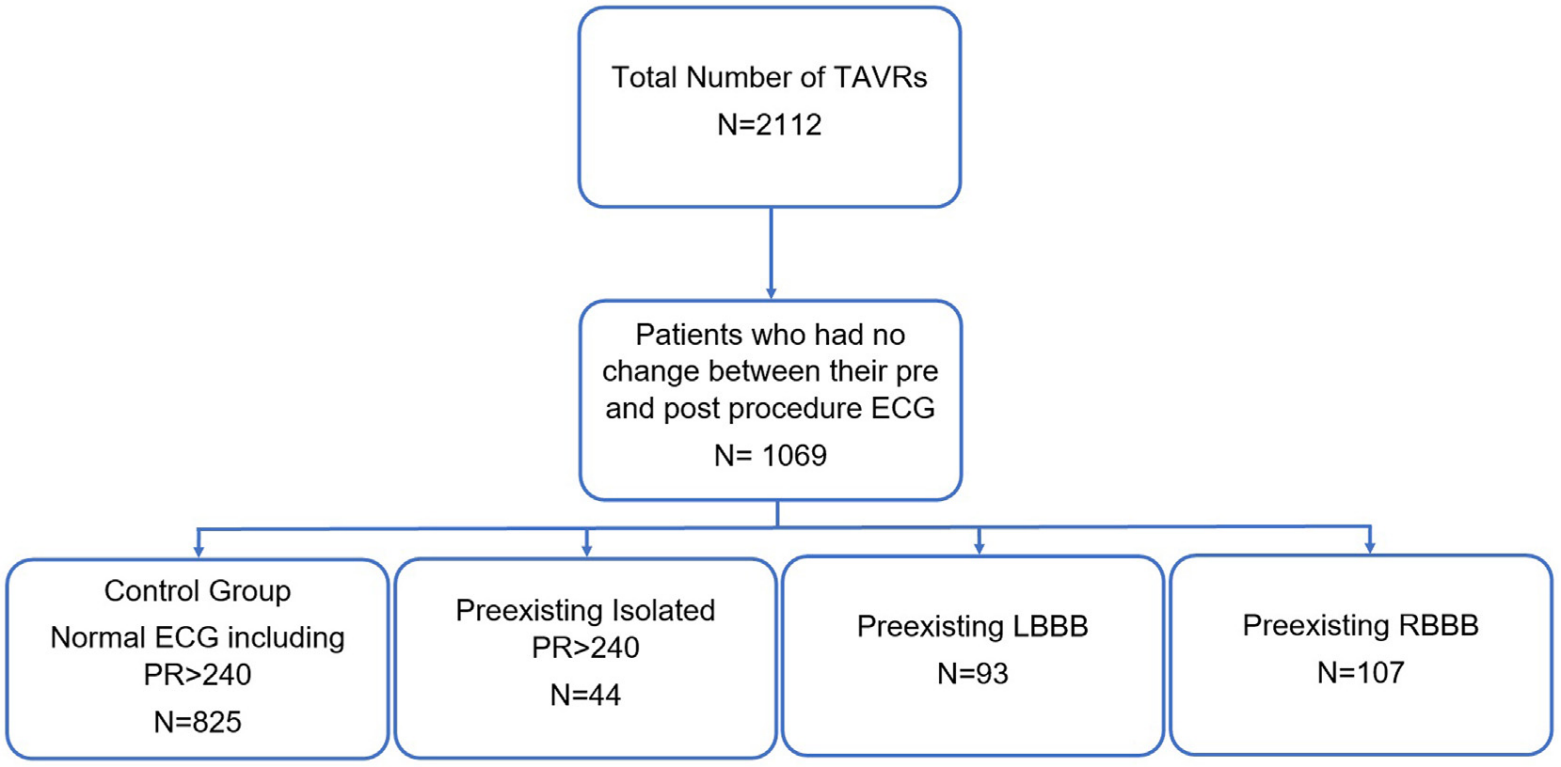
**Number of patients with unchanged normal and baseline conduction disease post-TAVR.** Flowsheet demonstrating the total number of patients who underwent TAVR between February 2012 and December 2021 and further stratified to demonstrate the number of patients in each of our study groups. ECG, electrocardiogram; LBBB, left bundle branch block; RBBB, right bundle branch block; TAVR, transcatheter aortic valve replacement.

A total of 1069 patients were included in the study. There were 825 patients in the control group, 44 patients had isolated PR of >240 milliseconds, 93 patients had LBBB, and 107 patients had RBBB at baseline (Figure 2). Baseline characteristics are outlined in Table 1. Female sex was more common in the control group when compared with the isolated PR group (46.7% vs 22.7%; *P* = .002) and the RBBB group (46.7% vs 35.5; *P* = .03). Patients with baseline conduction disease were also older when compared with the normal ECG group. Comorbidities were mostly similar when the control was compared with each of the other groups. The isolated PR interval >240 group had a higher incidence of previous coronary artery bypass grafting (36.4% vs 16.8%; *P* < .001) and end-stage renal disease requiring dialysis (6.8% vs 1.9%; *P* = .03). The LBBB group had a higher proportion of diabetes (26.9% vs 37.3%; *P* = .04) and atrial fibrillation or flutter (44.1% vs 33.1%; *P* = .03). The RBBB group had a higher proportion of previous coronary artery bypass grafting when compared with the control group (25.2% vs 16.8%; *P* = .03). Within the RBBB group, 15 (14%) patients had baseline LAFB and 1 (0.9%) had LPFB. Procedural characteristics are outlined in Table 2.

**Table 1:** Baseline characteristics.

	Normal ECG pre-TAVR and post-TAVR, including PR interval <240 ms (n = 825)	Isolated PR interval >240 ms (n = 44)	*P*^a^	LBBB (n = 93)	*P*	RBBB (n = 107)	*P*
Age, y	80 ± 11	83 ± 10	.04	83 ± 9	.004	82 ± 8	.005
Female sex	385 (46.7)	10 (22.7)	.002	46 (49.5)	.61	38 (35.5)	.03
Body mass index, kg/m^2^	28 ± 8	29 ± 7	.74	29 ± 6	.7	29 ± 8	.95
Hypertension	722 (87.5)	40 (90.9)	.5	86 (92.5)	.16	95 (88.8)	.71
Diabetes	308 (37.3)	18 (40.8)	.63	25 (26.9)	.04	47 (43.9)	.19
Current smoker	32 (3.9)	1 (2.3)	.59	1 (1.1)	.17	1 (0.9)	.12
ESRD on dialysis	16 (1.9)	3 (6.8)	.03	2 (2.2)	.89	2 (1.9)	.96
Previous stroke or TIA	126 (15.3)	4 (9.1)	.26	14 (15.1)	.96	12 (11.2)	.27
Atrial fibrillation/atrial flutter	272 (33.1)	8 (18.2)	.06	41 (44.1)	.03	34 (31.8)	.8
Coronary artery disease	527 (63.9)	31 (70.5)	.38	68 (73.1)	.07	69 (64.5)	.90
Previous PCI	315 (38.2)	17 (38.6)	.95	42 (45.2)	.19	41 (38.3)	.98
Previous CABG	139 (16.8)	16 (36.4)	<.001	18 (19.4)	.54	27 (25.2)	.03
STS score, %	4.3 ± 4.2	5.6 ± 5.8	.12	5.5 ± 7	.003	4.8 ± 5.4	.16
AV area, cm^2^	0.8 ± 0.21	0.9 ± 0.19	.02	0.8 ± 0.21	.47	0.86 ± 0.16	.18
AV mean gradient, mm Hg	46 ± 21	42 ± 10	.01	44 ± 16	.01	47 ± 22	.75
LVEF, %	63 ± 10	60 ± 12	.05	51 ± 23	<.001	64 ± 9	.18
NYHA Class							
I-II	301 (36.5)	16 (36.4)		28 (30.2)		44 (41.1)	
III-IV	524 (63.5)	28 (63.6)		65 (69.8)		63 (58.9)	

Values are mean ± SD or n (%). Mann-Whitney *U* test for continuous variables and χ^2^ test for categorical variables.

AV, aortic valve; CABG, coronary artery bypass graft; ECG, echocardiogram; ESRD, end-stage renal disease; LBBB, left bundle branch block; LVEF, left ventricular ejection fraction; NYHA, New York Heart Association; PCI, percutaneous coronary intervention; RBBB, right bundle branch block; STS, Society of Thoracic Surgeons; TAVR, transcatheter aortic valve replacement; TIA, transient ischemic attack.

a *P* values were calculated by comparing each of the groups with the control normal ECG pre-TAVR and post-TAVR group.

**Table 2:** Procedural characteristics.

	Normal ECG pre-TAVR and post-TAVR, including PR interval <240 ms (n = 825)	Isolated PR interval >240 ms (n = 44)	*P*^a^	LBBB (n = 93)	*P*	RBBB (n = 107)	*P*
Anesthesia							
General	263 (31.9)	15 (34.1)	.76	35 (37.6)	.26	34 (31.8)	.98
Moderate sedation	562 (68.1)	29 (65.9)		58 (62.4)		73 (68.2)	
Access site							
Femoral	721 (87.4)	37 (84.1)	.52	86 (92.5)	.15	95 (88.8)	.68
Transapical	82 (9.9)	6 (13.6)	.63	7 (7.5)	.46	10 (9.3)	.85
Other	22 (2.7)	1 (2.3)		0		2 (1.9)	
Valve type							
Balloon expandable	765 (92.7)	41 (93.2)	.91	82 (88.2)	.12	98 (91.6)	.67
Self-expanding	60 (7.3)	3 (6.8)		11 (11.8)		9 (8.4)	

Values are n (%). Mann-Whitney *U* test for continuous variables, and χ^2^ test for categorical variables.

ECG, echocardiogram; HAVB, high-grade atrioventricular block; LBBB, left bundle branch block; RBBB, right bundle branch block; TAVR, transcatheter aortic valve replacement.

a *P* values were calculated by comparing each of the groups with the control normal ECG pre-TAVR and post-TAVR group.

### Incidence of HAVB

After TAVR, the RBBB group had the highest incidence of HAVB within 30 days (17.8% vs 2.2% in the control group; *P* < .001). In the isolated PR interval >240 group and LBBB group, the incidence of HAVB tended to be higher but not statistically significant when compared with the control (4.5% vs 2.2%; *P* = .31, and 5.4% vs 2.2%; *P* = .06, respectively). All groups, except for the RBBB group, had a higher proportion of delayed HAVB occurring 24 hours after TAVR than within the first 24 hours (Table 3).

**Table 3:** Outcomes.

	Normal ECG Pre-TAVR and post-TAVR, including PR <240 ms (n = 825)	Isolated PR >240 ms (n = 44)	*P*^a^	LBBB (n = 93)	*P*	RBBB (n = 107)	*P*
Postprocedural HAVB within 30 d	18 (2.2)	2 (4.5)	.31	5 (5.4)	.06	19 (17.8)	<.001
Early HAVB (within 24 h post-TAVR)	5 (0.6)	0		1 (1.1)	.59	12 (11.2)	<.001
Delayed HAVB (>24 h post-TAVR)	13 (1.5)	2 (4.5)	.14	4 (4.3)	.06	7 (6.5)	<.001
Transient intraprocedural HAVB^b^	15 (1.8)	1 (2.3)		4 (4.3)		20 (19)	
Pacemaker implantation	50 (6.1)	4 (9.1)	.42	11 (11.8)	.03	42 (39.2)^c^	<.001
30-Day ambulatory ECG monitor	173 (21)	18 (40)		21 (23)		33 (31)	
30-Day mortality	4 (0.5)	1 (2.3)^d^	.14	0		3 (2.8)^e^	.009
1-Year mortality	57 (6.9)	1 (2.3)	.23	8 (8.6)	.55	11 (10.3)	.33

Values are n (%); χ^2^ test for categorical variables.

HAVB, high-grade atrioventricular block; LBBB, left bundle branch block; RBBB, right bundle branch block; TAVR, transcatheter aortic valve replacement.

a *P* values were calculated by comparing each of the groups with the control normal ECG pre-TAVR and post-TAVR group.

b This was defined as HAVB that developed shortly after valve deployment with recovery of native conduction before transfer to a monitored bed.

c Of 42 patients, 23 had PPM placed prophylactically, mostly for the indications of transient intraprocedural HAVB (6 patients) and presence of concomitant baseline LAFB with RBBB (10 patients).

d Cause of death was acute mesenteric ischemia.

e Two of the 3 patients who died had pacemakers placed after TAVR. The cause of death for all 3 cases is unclear.

### Pacemaker implantation and mortality

**Figure 3:**
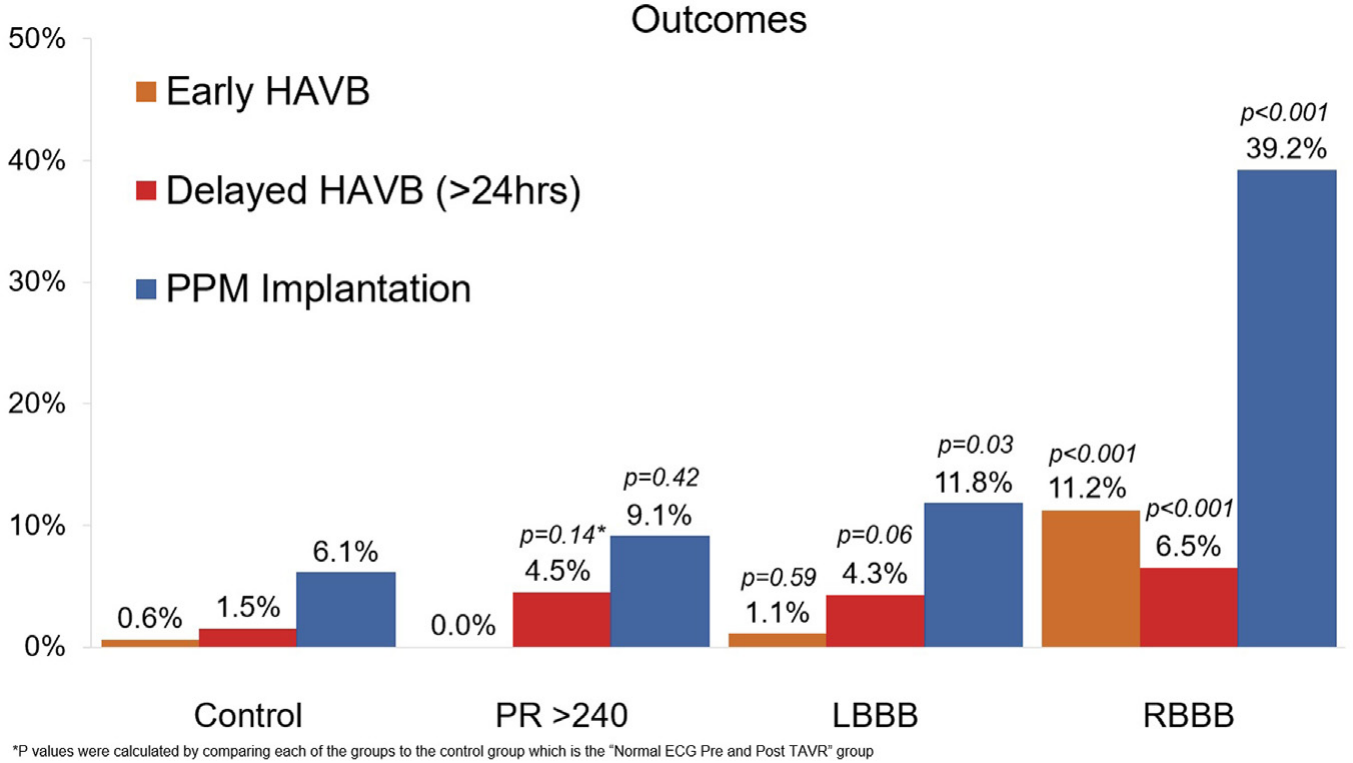
**Outcomes of patients with unchanged ECG post-TAVR.** Bar chart demonstrating the primary outcomes of early HAVB and delayed HAVB as well as PPM implantation rates in the study groups. *P* values were calculated by comparing each of the groups to the control normal ECG pre-TAVR and post-TAVR group. HAVB and PPM implantation was highest in the RBBB group. HAVB, high-grade atrioventricular block; LBBB, left bundle branch block; PPM, permanent pacemaker; RBBB, right bundle branch block.

Outcomes are summarized in Table 3 and Figure 3. The PPM implantation rate in the RBBB and LBBB group was higher when compared with that in the control (39.2% vs 6.1%; *P* < .001), (11.8% vs 6.1%; *P* = .03). The isolated PR interval >240 group and the normal ECG group had similar PPM implantation rate (9.1% vs 6.1%; *P* = .42). The 30-day mortality was highest in the RBBB group and was significantly higher than the control (2.8% vs 0.5%; *P* = .009). Two of the 3 patients who died in the RBBB group had pacemakers implanted. The cause of death for all 3 cases is unclear. There was 1 death in the isolated PR interval >240 group, which was due to acute mesenteric ischemia. There was no statistically significant difference in 1-year mortality when the 3 groups were compared with the normal ECG group.

### RBBB

**Figure 4:**
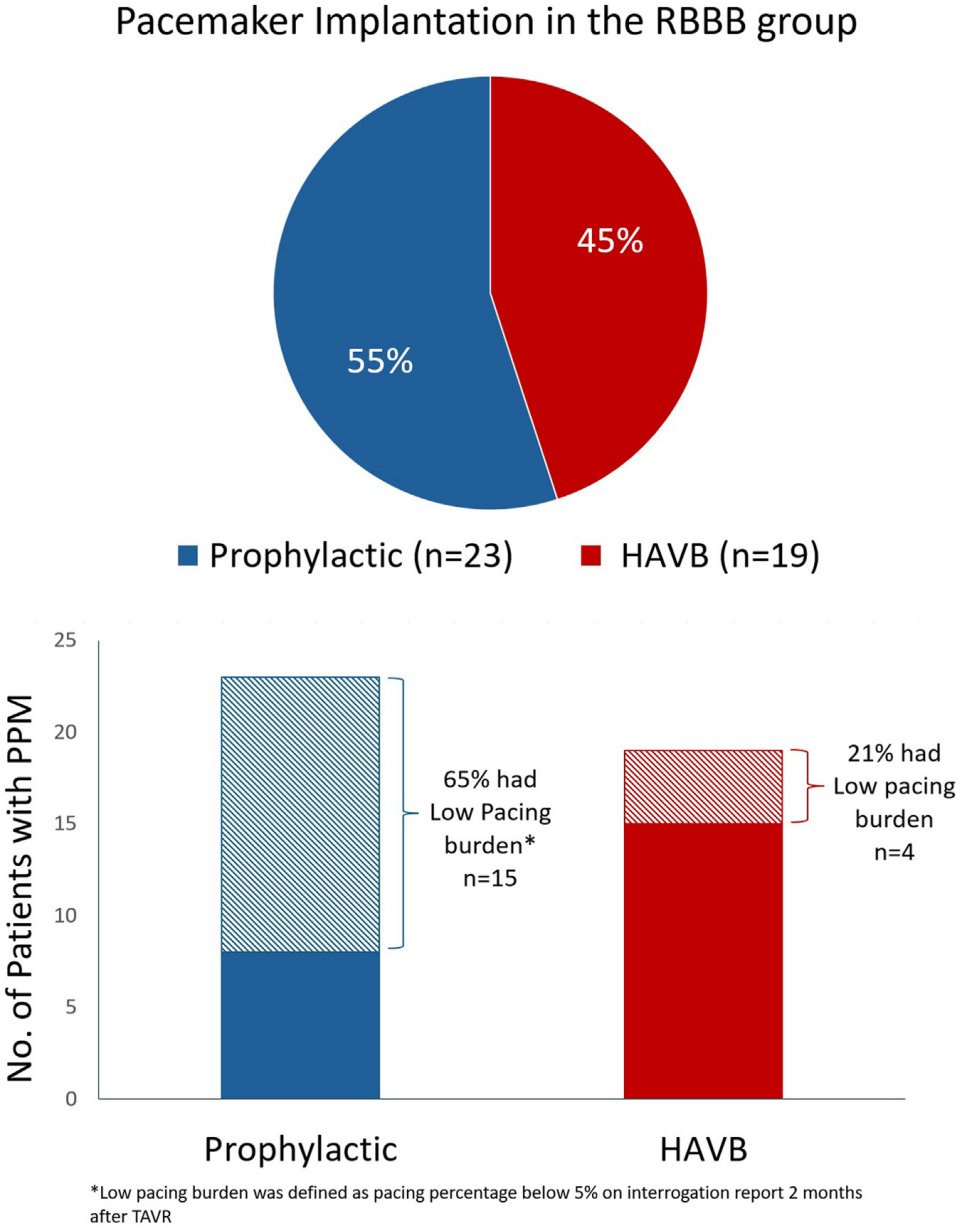
**Pacemaker implantation in the RBBB group.** A total of 42 patients in the RBBB group underwent PPM implantation. The indication for PPM implantation was HAVB in 45% of the patients; 55% of the patient in the group had PPM placed prophylactically with the 2 most common indications being the presence of baseline LAFB (10/23) and transient intraprocedural HAVB (6/23). Most patients (65%) who had prophylactic PPM had low pacing burden (<5%) on follow-up interrogation. HAVB, high-grade atrioventricular block; PPM, permanent pacemaker; RBBB, right bundle branch block.

In the RBBB group, 19 (17.8%) patients had postprocedural HAVB within 30 days of which 12 developed HAVB within 24 hours. Thirty-three patients in this group were discharged with a 30-day ambulatory ECG monitor. Seven of those patients developed HAVB and all events occurred within 1 week of discharge. PPM implantation occurred in 42 (39.2%) patients (Figure 4). Twenty-three patients had PPM implanted prophylactically. The most common indication for prophylactic PPM in the RBBB group was the presence of baseline LAFB (10/23) and second most common was transient intraprocedural HAVB (6/23). On review of PPM interrogation reports of the patients that had PPM placed prophylactically, the majority (15/23) had low right ventricular pacing frequency (<5%) at 2 months follow up. Indications for PPM and specific ECG characteristics were similar in the low pacing burden (<5%) patients when compared with patients who received prophylactic PPM but had a burden above 5%.

Logistic regression analysis was performed to identify predictors of HAVB in the RBBB group. All baseline clinical characteristics mentioned in Table 1 were tested as potential predictors of the primary outcome of HAVB within 30 days. Conduction abnormalities were also tested including first-degree AV block, LAFB, atrial fibrillation, transient intraprocedural HAVB, and post-TAVR QRS interval >150. Transient intraprocedural HAVB was the only significant risk factor in developing postprocedural HAVB (odds ratio [OR], 12.1; 95% CI, 3.9-37.8; *P* < .001).

### Left bundle branch block

In the LBBB group, only 5 (5.4%) patients developed HAVB within 30 days with 4 cases occurring more than 24 hours post-TAVR. The post-TAVR QRS interval was >150 milliseconds in 4 of the 5 patients who developed HAVB. Of the 21 patients in this group who were discharged with a 30-day ambulatory ECG monitor, only 1 developed HAVB. Eleven (11.8%) patients in the LBBB group had PPM implantation. Five patients had PPM placed prophylactically for the following indications: 3 for concomitant first-degree heart block with LBBB, 1 for transient intraprocedural heart block, and 1 for Mobitz type 2 second-degree AV block. Three out of 5 patients who received prophylactic PPM had a low pacing burden (<5%). The patient with Mobitz type 2 AV block had a pacing frequency of 98%. One patient with first-degree AV block combined with LBBB had a high pacing frequency (42%), although it is essential to note that this patient had a markedly prolonged PR interval of 242 milliseconds on their post-TAVR ECG. One patient in the LBBB received a single chamber PPM for the indication of sinus node dysfunction 3 weeks after TAVR.

### Isolated PR of >240

This group had only 2 patients (4.5%) who developed delayed HAVB and no patients developing early HAVB. Two out of the 4 patients who received PPM had it placed prophylactically due to progressive ECG changes. One of these patients had progressive lengthening of the PR interval to 388 milliseconds and had a pacing burden of 66%. The other patient developed a new LBBB on inpatient telemetry monitoring and had a pacing burden of 98%. Eighteen patients were discharged with 30-day ambulatory ECG monitoring, with only 1 patient developing HAVB on day 5 of monitoring.

## Discussion

This study investigated outcomes in patients with unchanged baseline conduction disease post-TAVR. We made the following observations: (1) Despite no immediate change in post-TAVR ECG in patients with pre-existing RBBB, 17.8% of patients developed postprocedural HAVB, mainly occurring within 24 hours, emphasizing the need for inpatient monitoring for at least 24 hours in this group. (2) Conversely, in patients with unchanged isolated PR interval >240 milliseconds or LBBB, the incidence of HAVB was relatively low with the majority occurring after 24 hours. (3) In the RBBB group, transient intraprocedural heart block strongly predicted developing postprocedural HAVB. (4) Most patients with pre-existing RBBB who had PPM placed prophylactically had low pacing burden (<5%).

Baseline RBBB is the strongest predictor of developing HAVB post-TAVR with multiple studies identifying ORs ranging from 13.16 to 45.1.^11^^,^^12^ Our study established that this remains true despite no new conduction abnormalities occurring immediately post-TAVR ECG. Our institutional algorithm places patients with pre-existing RBBB in either group 2 or 3 (Figure 1) depending on coexisting abnormalities and, therefore, managed with either ambulatory ECG monitoring or PPM implantation. In our cohort, delayed HAVB among patients discharged with 30-day ambulatory ECG monitor was 21%. All episodes of HAVB occurred within 1 week after discharge, suggesting that it may be reasonable to limit monitoring from 30 days to 2 weeks. The 2-week monitoring period is also supported by a prospective study by Muntané-Carol et al,^13^ which showed a rate 13.2% of delayed HAVB and zero 30-day mortality among patient with pre-existing RBBB discharged with 2 weeks of ambulatory ECG monitoring.^13^ The high rate of PPM implantation (39.2%) within the RBBB group can be attributed, in part, to the use of prophylactic PPM implantation based on our algorithm for managing this high-risk group. Approximately half of these patients underwent prophylactic PPM placement, contributing to the high implantation rate. The most common indication for prophylactic PPM was the presence of baseline LAFB. Most patients in our study who received the PPM prophylactically had low pacing burden (<5%). These patients could have avoided PPM implantation and instead managed by inpatient telemetry followed by ambulatory ECG monitoring. On the contrary, Fukutomi et al^14^ demonstrated that adopting a strategy of placing prophylactic PPM in patients with baseline RBBB and PR interval of >220 milliseconds can reduce the length of stay in the hospital and the rehospitalization for HAVB. We identified only transient intraprocedural HAVB as a significant predictor of HAVB in the RBBB group. We recommend that this group of patients with unchanged RBBB post-TAVR who are observed to have transient intraprocedural HAVB should be strongly considered for early PPM given this high rate of recurrence.

New LBBB post-TAVR is an established risk factor for developing HAVB with studies reporting incidence rates of approximately 15%.^15^^,^^16^ For pre-existing LBBB, Fischer et al,^17^ demonstrated an increased risk of PPM implantation when compared to no pre-existing LBBB (21.1% vs 14.8%). It is important to note that the authors could not stratify the data based on the indication of PPM implantation. Therefore, the higher PPM implantation rate observed may be driven by prophylactic pacing rather than the actual risk of HAVB. In contrast, our findings revealed that patients with baseline LBBB that is unchanged post-TAVR had a relatively low incidence of HAVB (5%), and majority of cases were delayed (>24 hours). This was also observed in the PR interval >240 group. El-Sabawi et al^11^ revealed pre-existing first-degree AV block was an independent risk factor for delayed HAVB (>24 hours) (OR 2.98 CI 1.39-6.38).^11^ In our cohort of patients with a PR interval >240 and no changes post-TAVR deployment, there were only 2 patients who developed HAVB, with both cases being after 24 hours. Our findings suggest that patients with pre-existing LBBB or PR interval >240 milliseconds that is unchanged post-TAVR could be considered for early dismissal with ambulatory ECG monitoring, given the low rates of HAVB and with events mainly being delayed.

The 30-day mortality was higher in the RBBB group compared with the control; however, it is essential to note that 2 of the 3 patients who died had PPM implantation for HAVB. We did not have the details for one of the deaths and, therefore, cannot establish whether it was due to HAVB or complete heart block. The 1-year mortality tended to be higher in the RBBB compared with the other groups, consistent with findings in other studies that demonstrated RBBB is associated with increased mortality.^5^^,^^18^ Importantly, this association does not imply that the RBBB itself is a cause of increased mortality, particularly given differences in baseline comorbidities observed with RBBB.

### Limitations

A significant limitation of this study includes its retrospective observational design. Another limitation is that our institutional algorithm used in the analysis was implemented for clinical use in April 2020, however, our data comprised patients from 2012 to 2021. This resulted in variability within the groups in patients undergoing 30-day ambulatory ECG monitoring or PPM implantation. Additionally, our algorithm recommends 30-day ambulatory ECG monitoring for both patients with LBBB and isolated PR >240 intervals. This may lead to selection bias in detecting delayed HAVB, especially in the isolated PR interval >240-millisecond group as 40% of these received extended ECG monitoring compared with 21% in the control. Most of the patients in this study had balloon-expandable valves, limiting the study’s generalizability to patients undergoing self-expandable valves.

## Conclusions

This study provides valuable insights that can contribute to refining clinical decision making and optimizing patient care pathways for the management of conduction system disease associated with TAVR. While patients with pre-existing RBBB exhibited a substantial risk of developing HAVB despite stable post-TAVR ECGs, those with unchanged LBBB or prolonged PR interval of >240 milliseconds had relatively lower incidences of HAVB, predominantly occurring beyond the initial 24-hour period, allowing for early hospital dismissal (Central Illustration).

**Central Illustration:**
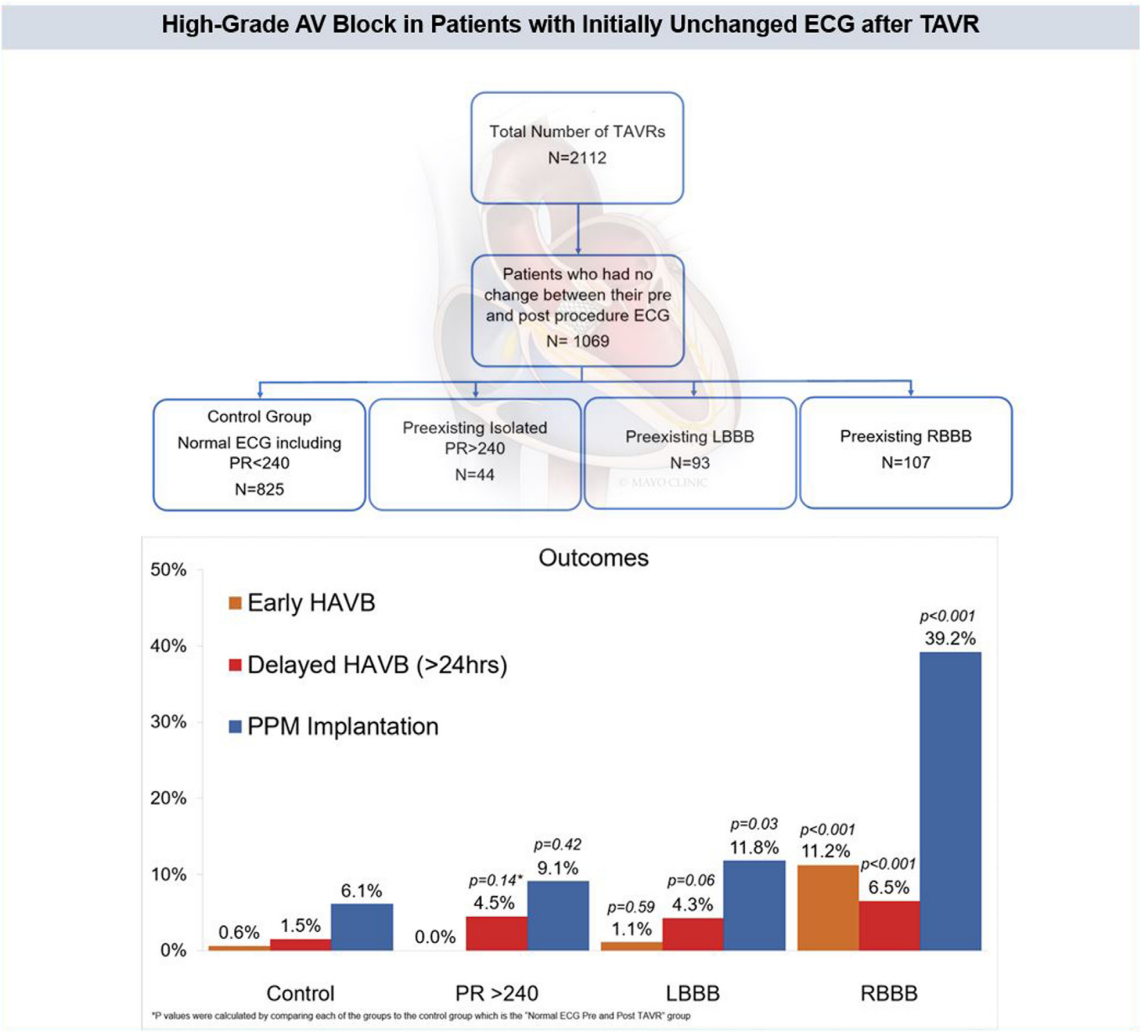
**High-grade AV block in patients with initially unchanged ECGpost-TAVR.** Among 2112 patients who underwent TAVR, 1069 patients had no change between their preprocedural and postprocedural ECG. Patients with pre-existing and unchanged first-degree heart block (PR > 240), LBBB, and RBBB were compared with patients with normal ECG. HAVB, delayed HAVB (>24 hours), and PPM implantation were highest in the RBBB group and statistically significant when compared with the normal group (39.2% vs 6.1%, 6.5% vs 1.5% and 11.2% vs 0.6%, respectively). Patients with unchanged LBBB or prolonged PR interval of >240 had relatively lower incidences of HAVB, predominantly occurring beyond the initial 24-hour period. HAVB, high-grade atrioventricular block; LBBB, left bundle branch block; RBBB, right bundle branch block; PPM, permanent pacemaker; TAVR, transcatheter aortic valve replacement.
